# Assessing the performance of only HRP2 and HRP2 with pLDH based rapid diagnostic tests for the diagnosis of malaria in middle Ghana, Africa

**DOI:** 10.1371/journal.pone.0203524

**Published:** 2018-09-07

**Authors:** Dennis Adu-Gyasi, Kwaku Poku Asante, Sabastina Amoako, Nicholas Amoako, Love Ankrah, David Dosoo, Samuel Kofi Tchum, George Adjei, Oscar Agyei, Seeba Amenga-Etego, Seth Owusu-Agyei

**Affiliations:** 1 Kintampo Health Research Centre, Ghana Health Service, Kintampo, Brong Ahafo, Ghana; 2 Institute of Health Research, University of Health & Allied Sciences, Ho, Ghana; Academic Medical Centre, NETHERLANDS

## Abstract

**Background:**

Rapid diagnostic test (RDT) kits have been useful tools to screen for the presence of infection with malaria parasites. Despite the improvement, false results from RDTs present a greater challenge. The need for quality test kits is desirable. We evaluated the diagnostic accuracy of three malaria RDTs.

**Method:**

The team consented and enrolled 754 participants from the two major public hospitals in Kintampo districts of Ghana from June 2014 to August 2014. Venous blood samples were obtained by trained personnel and samples were screened for malaria using CareStart and SD Bioline HRP2 and HRP2 with pLDH based RDTs with blood slides for malaria microscopy as “gold standard”. Geometric mean parasite densities were estimated and parasite densities were used to estimate the quantitative limits of the RDTs. The sensitivities, specificities and other performance criteria were calculated using statistical analytical software.

**Result:**

The median age of participants was 21 (range 5–31) years. There were 28.6% (215/752) were males and 71.4% (537/752) were females. Comparing with microscopy, the sensitivity, specificity, positive predictive value, negative predictive value and the ROC were for CareStart (HRP2), 98.2%, 66.5%, 82.6%, 95.6%, 0.82; for CareStart (HRP2/pLDH) 98.2%, 66.5%, 82.6%, 95.6%, 0.82 and for SD-Bioline (HRP2/pLDH) RDTs 98.2%, 69.2%, 84.2%, 96.0%, 0.84 respectively. The performance for all the kits were acceptable at a cut-off of 25 or more parasites/μl of blood.

**Conclusions:**

The diagnostic performance of the three malaria RDTs was acceptable, according to the World Health Organisation criteria, to detect densities ≥25 parasite/μl of blood. The RDTs with HRP2/pLDH targets were comparable to those with only HRP2 and could successfully substitute current and commonly used HRP2-based RDTs.

## Background

Malaria parasite infection contributes greatly to morbidity and mortality in the developing part of the world [1]. A larger proportion of the cases of malaria infection in Africa is attributable to *Plasmodium falciparum* infection [2, 3]. *Plasmodium falciparum (Pf)* is responsible for the high cause of deaths in children who are 5 years of age or less [4, 5]. Malaria is typically diagnosed using light microscopy and this is accepted and regarded as the reference method [6–8]. A number of rapid diagnostic tests (RDTs) kits have also been developed for use in both non-endemic and endemic countries as part of malaria management and control programs [9] and the quality of kits produced has improved over the years [1, 10].

Artemisinin-based Combination Therapy (ACT) use, as recommended by the World Health Organisation (WHO), is restricted to patients with confirmed malaria parasite infections [11, 12]. With the challenge of availability of skill and logistics to microscopy in several facilities in sub-Saharan Africa, RDTs are used for confirmation of malaria parasite infection during diagnosis before treatment with antimalarials. In the absence of RDTs, most febrile illnesses are treated presumptively [13] as malaria without laboratory confirmation and especially where the skill for microscopy does not exist. The effects of presumptive diagnosis and treatment such as increased cost of treating malaria, missing other diagnosis, extensive overuse of anti-malarial have extensively been published [14]. It is therefore expedient to make available an economic and time efficient method that is reliable but requires less skills to perform. This gap is filled by introduction of quality RDTs [10, 15, 16]. Conversely, RDTs could give false results [8, 17] which present another challenge. The reason is that initiation of treatment might be delayed and this may possibly lead to deterioration in the condition of victims with malaria. The quality and performance of RDTs is not in doubt [18]. RDTs have improved from the start of their production and the product testing programme by WHO and the Global Health in 2017 [1, 10, 18]. In a study carried out in Ghana, the performance of an Histidine-rich protein 2 (HRP2)-based RDTs recorded a sensitivity and specificity of 100% and 73% respectively [7]. In Tanzania and among children (2 months to 13 years), testing of HRP2 branded RDT revealed a sensitivity of 98.2% [19]. Of great concerns were the false-negative results reported in other studies. In one, RDTs missed 8.8% (3/34) of malaria parasite positive cases in which parasitaemia were above determined detectable thresholds of the RDTs [20]. Histidine-rich protein 2 (HRP2) target is used for most malaria RDTs and the test is based on detection of the protein expressed by the *Plasmodium falciparum hrp2* gene [8]. Spontaneous *hrp2* deletions is reported to occur as was seen by Koita et al, 2012 and that *P*. *falciparum* parasites with such deletions cannot be detectable [8, 17] using RDT targeting HRP2 protein. RDTs based solely on HRP2 would give a false-negative results when used to screen infected persons with HRP2 deleted strains of malaria parasites. With the quest to improve malaria diagnostic testing and to overcome some of the anticipated challenges infection with parasites with *hrp2*-deleted gene pose, combination of multiple protein targets is suggested in the development of malaria RDTs. One of such parasite protein is the parasite Lactate Dehydrogenase (pLDH). It is believed that addition of pLDH to HRP2 in RDTs can improve their performance by identifying malaria parasites with *hrp2-gene* deletion [17, 18], help to differentiate current from convalescent infections and even to possibly identify infections that persist as a result of treatment failure [18, 21, 22]. We evaluated the diagnostic accuracy of CareStart (HRP2), CareStart (HRP2-pLDH) and SD-Bioline (HRP2/pLDH) using light microscopy as “gold standard” in middle Ghana, Africa among hospital attendant participants.

## Methods

### Ethical statement

The team presented all information including the objectives and procedures of the study to every participant. The team sought and obtained written informed consent from all adult participants and from the parents of all minors to voluntarily participate in the study. Care-takers were invited to give their written informed consent on behalf of participating minors. Children aged 10 to 17 years were invited to give their written informed assent for consenting in addition to their parental consent. The Kintampo Health Research Centre Institutional Ethics Committee (KHRC-IEC) (FWA No. 00011103) gave ethical approval of the protocol before the study commenced.

### Study design, site, population and participant selection

The team adopted a cross-sectional study design. The study was conducted in Kintampo North Municipal and Kintampo South District all in the Brong Ahafo Region of Ghana, West Africa. The study area is estimated to cover about 7162 sqkm with a resident population of approximately 140,000 [23]. The location of the study area has been well described by Owusu-Agyei et al 2009 [23, 24]. Prevalence of malaria parasitaemia (symptomatic/asymptomatic) in the area was about 50% among children below 10 years old. The area had entomological inoculation rate (EIR) of 269 infective bites per person per year [24–26] and malaria transmission is perennial. There are three (3) hospitals, more than twelve (12) clinics [27] and 30 community-based health planning services (CHPS) compounds [28]. Participants were recruited from the two major public hospitals that serve as patient referral points in the study area. In these facilities, routine malaria diagnosis is made either by malaria microscopy or using Rapid Diagnostic Test (RDT) kits. In Community based health facilities (CHPS compounds), RDTs are mainly used to diagnose malaria parasite infection.

### Sample size determination

A minimum sample size of 350 was determined, based on our sample size calculation. We assumed 0.98 expected Area under ROC curve compared to 0.95 of a Null hypothesis value [7], 1:2 ratio of sample sizes in negative/positive groups and 5% confidence limits. At 90% power, a minimum of 233 malaria-positive and 117 malaria-negative participants were considered to be enough to evaluate the accuracy of the malaria RDTs kits in this study (MedCalc version 12.5 software, Mariakerke, Belgium).

### Study procedure

Personnel from Kintampo Health Research Centre (KHRC) and the two major public hospitals carried out the study activities to recruit the number of participants needed from the health facilities from June 2014 to August 2014. Patients that had requests on their forms for malaria tests to be done in the hospitals’ laboratories were contacted to seek their voluntary written informed consent to be recruited to participate in the study. The study required participants to donate 2mls of venous blood samples for evaluation of the test kits. The study information, activities and procedures were explained in turn to each participant; allowing each of them time to decide whether to participate or not. The study team obtained written informed consent from each participant enrolled in the study period from the two major public hospitals. Demographic data such as age and sex were collected. The blood samples were collected into EDTA test tubes by trained study laboratory personnel and transported to the KHRC laboratory for analysis according to the Standard Operating Procedures for sample collection, transport and analysis. Ten percent (10%) of the samples were retested as quality assurance measure for the RDT methods. This was done by selecting every 10th sample for RDT retesting.

### Laboratory processes

Blood samples taken from the two major health facilities and received in KHRC laboratory were used to screen for malaria parasites using the three malaria RDTs (CareStart Malaria (HRP2), CareStart Malaria (HRP2/pLDH), SD Bioline Malaria (HRP2/pLDH)) and also using routine microscopy as the “gold standard”.

#### Blood slide reading for malaria parasite

Thick and thin blood slides films were prepared from each sample received in the KHRC laboratory and used for malaria parasite identification as described previously [24, 29]. In brief, a measured volume of 6 μl of blood was used for the thick film on a predefined template of 12 mm and 2 μl for the thin film. For 10 minutes, the slides were stained with 10% (1:9 ml) of fresh working solution of Giemsa stains prepared freshly prior to staining the thin and thick blood smears after fixing the thin smear with absolute methanol [24, 29]. Two competent (grading based on National Institute for Communicable Disease Assessment, South Africa), malaria microscopists independently read the blood smears [24, 29]. A third reader was used whenever there was discordance between the first two microscopists. Final results were produced from two results that agreed from two of the three independent microscopists.

#### Malaria Rapid Diagnostic Test (RDT) Kits

Participants’ samples were also screened for malaria parasites using 5 μl with each of the RDTs from CareStart Malaria (HRP2), CareStart Malaria (HRP2/pLDH) (both manufactured by Access Bio Inc., New Jersey USA) and SD Bioline Malaria Antigen Pf (HRP2/pLDH) (Standard Diagnostics, Hagal-Dong, Korea). Each of the CareStart malaria RDTs (both HRP2 and HRP2/pLDH) had only two bands consisting of a control (“C”) and test (“T”) lines each. With the CareStart RDTs, a test is positive for malaria parasite when there is a reaction in the control and test lines. The control band must at all times be positive before a test could be classified as valid (Manufacturer’s instructions). For SD Bioline Malaria Antigen *Pf* (HRP2/pLDH), there were three bands representing a control line (“C”) and two separate test lines (labelled as “T1” and “T2”). The antigens that were targeted were *Pf*-HRP2 (T1) and *Plasmodium vivax* (Pv)-, *Plasmodium ovale* (Po)-, *Plasmodium malariae* (Pm)-or *Pf*-pLDH (T2) put on the same strip [22]. For the interpretation of the SD Bioline test kit as appeared in the packaging insert, two colour band, “C” and “T1” indicated *Pf* positive while “C” and “T2” indicated positive test for other *Plasmodium* species (*Pv*., *Pm*, *Po* or *Pf*). With three colour bands, positive “C”, “T2” and “T1” lines were interpreted as mixed infection of *Pf* and any of the other *Plasmodium* species. There was no case where “T1” was negative and “T2” was positive. The study strictly adhered to the manufacturers’ instructions to obtain good results from the RDTs. Results from two independent observers that were concordant were used as the final results for the RDT tests. One general challenge that was identified with both RDTs was the fact that extra caution needed to be taken to be able to fill the volume of blood needed for testing to the mark (5 μl). Improving in the process of filling the capillary tubes would improve in the test performance time.

#### Measurement of blood cell counts

Full Blood Count of haematological parameters was determined with ABX Micros 60 analyzers (Horiba-ABX, Montpellier, France). The validated instrument was used to obtain the counts of blood cells of interest to estimate the malaria parasite densities [24, 29–31] necessary for the evaluation studies.

## Data management and analysis

Supervisors checked for data completeness and consistency. Data were then double-entered using Microsoft Access software 2010. All queries were resolved after data entry. Stata Software version 13 (Stata Corporation, TX USA) and MedCalc software version 12.5 (Mariakerke, Belgium) were used on the cleaned data for analysis. The calculated sensitivity (SE), specificity (SP), negative and positive predictive values (PPV and NPV) and the Area under the Receiver Operator Characteristics (ROC) curve using malaria microscopy results as “gold standard” was used to estimate the diagnostic performance of the malaria RDTs. In estimating SE, we calculated the proportion of positive test results identified by the test kit compared to the positives given by the “gold standard”. With SP of the RDT, the proportion of negative test results was compared to the true negatives given by the “gold standard” (microscopy). The PPVs and the NPVs were calculated as described by Endeshaw et al, 2012 [32]. All reported parameters in the study were considered significant with 95% Confidence Intervals.

The formulae below were employed in our calculations.

$$Sensitivity=\frac{Number\;of\;true\;positives\;(TP)using\;the\;reference\;method}{\left(Number\;of\;true\;positives\;(TP))+\;(Number\;of\;false\;negatives\;(FN)\right)}$$

$$Specificity=\frac{Number\;of\;true\;negatives\;(TN)\;using\;the\;reference\;method}{\left(Number\;of\;true\;negatives\;(TN))\;+\;(Number\;of\;false\;positives\;(FP)\right)}$$

$$False\;Positive\;Rate\;(FPR)=\frac{False\;Positive(FP)}{\left(False\;Positive\;(FP))\;+\;(True\;Negative\;(TN)\right)}$$

## Quality assurance measures adopted

**Fig 1:**
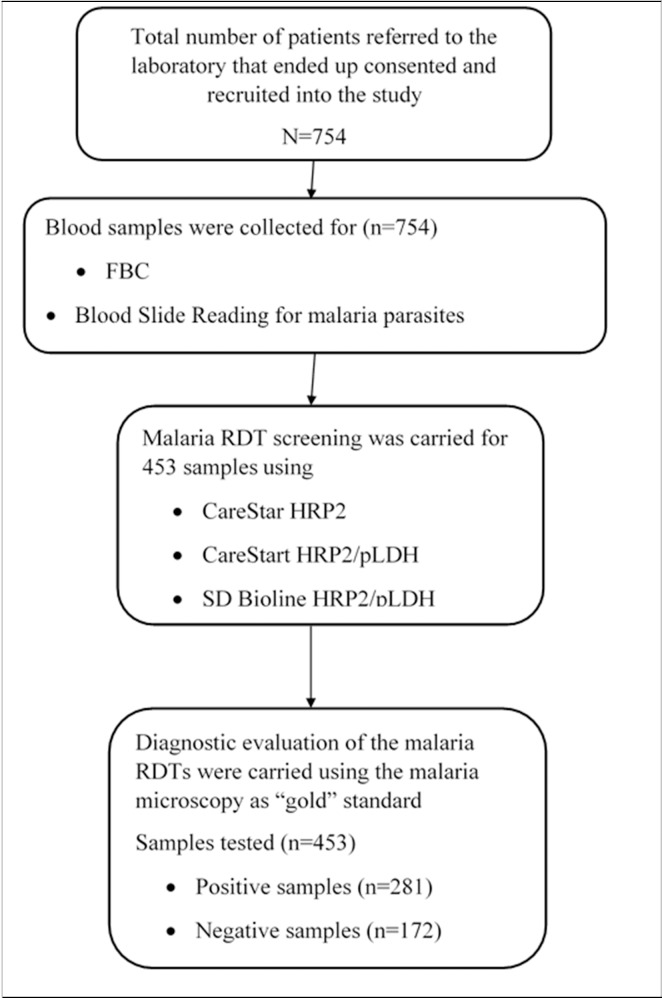
Flow chart for participant recruitment and data analysis.

All personnel that were involved in the study and testing processes before the start of the study were trained on the protocol and procedures. According to, and using the manufacturers’ instructions, the training included handling the RDTs, picking blood with the pipette for testing on the RDTs, use of the buffer for RDT and the reading and interpretation of test from the RDTs. The methods for preparing the blood films (thin and thick), staining and species identification were also included in the training schedule [24, 29]. The team also included training personnel to uniformly classify test outcomes as ‘‘positive” or ‘‘negative” as described in the manufacturers’ instructions for the RDTs. The team that performed the tests were blinded to the processes and results of each method of other tests. Quality controls for blood count parameters were performed with commercially available controls from Horiba-ABX, Montpellier, France but already prepared “Positive” and “Negative” microscope slides were used to quality control the giemsa working stains [30, 31] for each day’s work. The malaria RDT kits were controlled internally with known positive and negative samples from previous days’ work. The flow chart shown in Fig 1 gives the number of recruited participants’ and samples analysed.

## Results

### Study population characteristics

A total of 453 out of 754 samples from participants recruited from the two major public hospitals in the Kintampo North and South districts were used to evaluate the rapid diagnostic test kits. Of these, 28.7% (130/453) were males and 71.3% (323/453) were females. The demographic profile of the participants are shown in Table 1. The median age of participants was 21 (5–31) years. The prevalence of malaria based on microscopy in the number of participants recruited into the study was 39.1% (281/754) (Fig 1). The rank sum test for malaria parasite densities compared among males (n = 216) and females (n = 538) participants recruited for the study were similar (p = 0.218).

**Table 1:** Demographic profile of the population involved in this study.

Sex		n (%)
	Males	216 (28.7)
	Females	538 (71.3)
**Age Group (years):**		n (%)
	<11	183 (40.4%)
	11–20	68 (15.0%)
	21–30	113 (24.9%)
	31–40	49 (10.8%)
	>40	40 (8.8%)
**Any antimalaria in the last week**		n (%)
	Yes	105 (14.1)
	No	642 (85.9)

### Comparing the diagnostic accuracy of malaria RDTs and microscopy (“gold standard”)

The parasite density for *P*. *falciparum* ranged between 25 and 903120 parasites/μl of blood (IQR: 589–88580) with median density of 9695 parasite/μl.

**CareStart malaria (HRP2) RDT and Malaria microscopy:** The SE, SP, PPV and NPV with their confidence limits, of CareStart malaria (HRP2) RDT method compared to the “gold standard” were 98.2% (95% CI: 95.9, 99.4), 66.3% (95% CI: 58.7, 73.3), 82.6% (95% CI: 78.1, 86.5) and 95.8% (95% CI: 90.5, 98.6) respectively (Table 2). The ROC was 0.82 (95% CI: 0.79, 0.86) (Table 2). The kit reported a false positive rate (FPR) of 34% for true disease and a false negative rate of 1.8%.

**Table 2:** Diagnostic performance of the *CareStart* malaria (HRP2 and HRP2/pLDH) and SD bioline malaria (HRP2/pLDH) RDT kit to microscopy.

	Gold standard Positive (N = 281)		Gold standard Negative (N = 172)		Sensitivity % (95% CI)	Specificity % (95% CI)	PPV % (95% CI)	NPV % (95% CI)	AUC (95% CI)
	RDT Positive (n)	RDT Negative (n)	RDT Positive (n)	RDT Negative (n)					
Type of Malaria RDT									
*CareStart* (HRP2)	276	5	58	114	98.2 (95.9, 99.4)	66.3 (58.7, 73.3)	82.6 (78.1, 86.5)	95.8 (90.5, 98.6)	0.82 (0.79, 0.86)
*CareStart* (HRP2/pLDH)	276	5	58	114	98.2 (95.9, 99.4)	66.3 (58.7, 73.3)	82.6 (78.1, 86.5)	95.8 (90.5, 98.6)	0.82 (0.79, 0.86)
SD Bioline (HRP2/pLDH)	276	5	53	119	98.2 (95.9, 99.4)	69.2 (61.7, 76.0)	83.9 (79.5, 87.7)	96.0 (90.8, 98.7)	0.84 (0.80, 0.87)

#### CareStart malaria (HRP2/pLDH) RDT and Malaria microscopy

The SE, SP, PPV and NPV with their confidence limits, of CareStart malaria (HRP2/pLDH) RDT method compared to the “gold standard” were 98.2% (95% CI: 95.9, 99.4), 66.3% (95% CI: 58.7, 73.3), 82.6% (95% CI: 78.1, 86.5) and 95.8% (95% CI: 90.5, 98.6) respectively (Table 2). The ROC was 0.82 (95% CI: 0.79, 0.86) (Table 2). The kit reported a false positive rate (FPR) of 34% for true disease and a false negative rate of 1.8%.

#### SD Bioline malaria (HRP2/pLDH) RDT and Malaria microscopy

The SE, SP, PPV and NPV with their confidence limits, of SD Bioline malaria (HRP2/pLDH) RDT method compared to the “gold standard” were 98.2% (95% CI: 95.9, 99.4), 69.2% (95% CI: 61.7, 76.0), 83.9% (95% CI: 79.5, 87.7) and 90.4% (95% CI: 90.8, 98.7) (Table 2). The ROC was 0.84 (95% CI: 0.80, 0.87) respectively (Table 2). The kit reported a false positive rate (FPR) of 31% for true disease and a false negative rate of 1.8%.

In 5 samples, all three RDTs read negative compared to the “gold standard”. The parasite densities in these 5 samples were 36, 44, 36, 73 and 81 parasites/μl of blood. However, the RDTs could identify positive cases compared to microscopy for 19 other samples with parasite densities from 25 parasites/μl of blood to 96 parasites/μl of blood. This is suggestive of something else other than the low parasite density which might have led to the 5 negative results in Table 2. Again, none of the 5 participants claimed they had not taken antimalarial in the last week prior to our visit.

#### Performance of malaria RDTs at parasite density cut-off points

**Fig 2:**
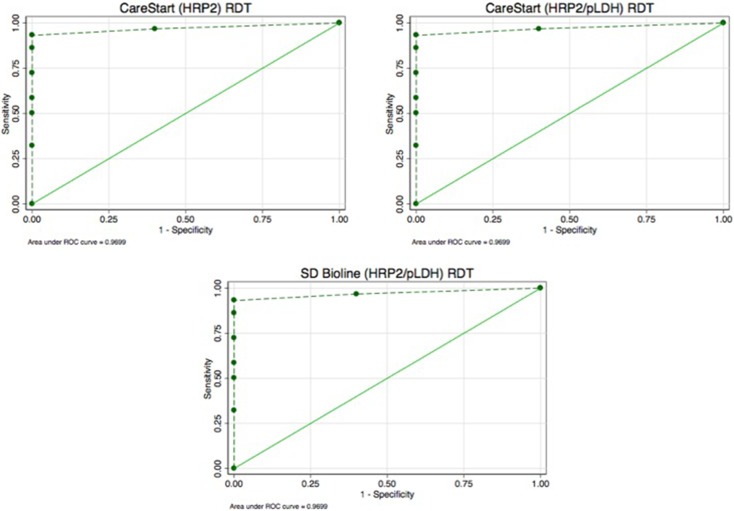
ROC curve of the three malaria RDT performance with plotted points of parasite densities at selected cut-off points.

Considering the performance of the kit at a cut-off criteria of 25, 50, 100, 200, 1000, 5000, 10000, 50000 and 1000000 parasites/μl of blood, a ROC of 0.97 was obtained for all the three malaria RDTs as presented in Fig 2. The sensitivities of the RDT kits were high at malaria parasite densities from 25 parasite/μl to 200 parasites/μl of blood (S1–S3 Tables). The agreement between CareStart Malaria (HRP2) and CareStart Malaria (HRP2/pLDH) was high (k = 100%) and either CareStart Malaria (HRP2) or CareStart Malaria (HRP2/pLDH) compared with SD Bioline Malaria Antigen P.f (HRP2/pLDH) also had high agreement (k = 98.7%) each.

## Discussion

Rapid Diagnostic Tests (RDTs) of different makes have played significant roles in the management of malaria. As new formulations emerge, they need to be assessed to determine their roles in malaria diagnosis. The diagnostic performances of CareStart Malaria (HRP2) RDTs compared to malaria microscopy, of CareStart Malaria (HRP2/pLDH) RDTs, and of SD Bioline Malaria Antigen *Pf* (HRP2/pLDH) RDTs evaluated in this study were acceptable with sensitivities higher than 98% and varied specificity from 66.3% to 69.2% (Table 2). The accuracy recorded in this study is comparable to what have been documented by other studies [7, 22].

The diagnosis, treatment and overall management of malaria adopt several guidelines using clinical judgement together with the parasitological detection of malaria parasites based on available accurate, validated and accepted standards for testing. Malaria which is known to be a leading cause of morbidity and mortality in the developing world [1] is preferably diagnosed using malaria microscopy as the “gold standard”. The quality of equipment, quality assurance measures, time required and level of expertise required to effectively produce reliable results using microscopy [29] has always been a challenge in resource deprived settings and largely in the developing world. It has been the expectations that malaria RDTs could fill the gap in those deprived settings to enable rapid screening and aid in malaria diagnosis. It is interesting to note that performance of some of the malaria RDTs including those evaluated in this study are comparable to using the microscope [7] despite their limitations in sensitivity and specificity [8, 22, 32]. One of such limitations is that some of the RDTs just as those evaluated in this study could have false positive rates >10% [1, 18, 21] and need to be looked at since such kits have increased possibility of giving false positive result.

The technique employed in developing these RDTs is based on detecting the HRP2 or HRP2 with pLDH proteins of malaria parasites if present. Recent reports of human infections with malaria parasites with *hrp2 gene deletion* have brought up the discussion of “false negative reporting” when RDTs solely developed with HRP2 based proteins are used for testing [8, 17]. It is worth mentioning that the performance of the three brands of malaria RDTs evaluated in this study had high diagnostic performance based on sensitivity according to acceptable standards of WHO (Fig 2 and Table 2) just as those solely developed with HRP2 targets. This suggests that RDTs developed to target both HRP2 and pLDH antigens simultaneously perform as well as those manufactured to detect only the HRP2 antigens of the malaria parasites. The RDTs could be used for screening and diagnosis when there is the fear of reduced reliability of HRP2-detecting RDTs because of *hrp2 gene deletion* in some strains of malaria parasites [33]. This is on the assumption that parasites with *hrp2 gene deletion* are expected to produce pLDH which would be detected when the parasite is present considering the time of sample analysis [21, 22]. However, the possibility of the RDTs being able to detect parasites with *hrp2 gene deletion* needs to be explored. This is because, the RDTs could not identify the infection that was identified by microscopy in 5 samples. In these samples it is unlikely that the RDTs failed to identify parasites because they had low parasite density since other samples had comparable parasite densities but were correctly identified by the RDTs. A thorough review of HRP2-based RDTs is required given the reports of *hrp2 gene deletion* infections in Mali [8] and potentially in Ghana as suggested by Amoah et al, 2016 [17].

The three RDTs evaluated in this study had acceptable performance in samples that had parasite densities from 25 to 200 parasites/μl of blood. It is assuring to note that the kits with targets to pLDH detecting antigens in addition to HRP2 performed well compared with only HRP2 antigen detecting kit at low parasite densities. The RDT kits particularly with pLDH detecting antigens can therefore be considered for use to screen and detect infections with *Plasmodium falciparum*. The kits therefore have the potential to be used for effective malaria diagnosis that will prevent over prescription of antimalarials.

These malaria RDTs which are all packaged ready for use require less skills and complex equipment to perform. The malaria RDTs evaluated in this study like all others used approximately 15 minutes to give reliable results without any equipment that require electricity. This will effectively improve early detection and diagnosis of malaria parasite infection for prompt treatment especially in endemic region.

### Limitations of the study

The study was carried out in three months (June-August), corresponding to the rainy season where malaria transmission is very high. However, malaria transmission and infection is perennial with peaks and troughs in the study area and the range of parasite densities recorded in this study cut across low to high densities. It is assumed that results obtained from the rainy season would apply in the dry season as well, which may not be true. However, the sample size considered adequate number of positive and negative cases in their correct ratios and that in our view would not be significantly affected by season in validating the results and performance of the kits.

### Conclusion

The diagnostic performance of the HRP2 and HRP2 with pLDH based RDTs assessed in this study was acceptable and could detect parasitaemia as low as 25 parasite/μl of blood. The HRP2 RDTs combined with pLDH targets be considered for malaria control programmes. There is however a decrease in specificity which could result from the persistence of HRP2 proteins even weeks after effective treatment of malaria.

## Supporting information

S1 Table Diagnostic performance of the *CareStart* malaria (HRP2) RDT kit to microscopy exploring parasite density cut-off points. (DOCX)

S2 Table Diagnostic performance of the *CareStart* malaria (HRP2/pLDH) RDT kit to microscopy exploring parasite density cut-off points. (DOCX)

S3 Table Diagnostic performance of the SD Bioline malaria (HRP2/pLDH) RDT kit to microscopy exploring parasite density cut-off points. (DOCX)
