# Supplementary material for: Assessing the performance of only HRP2 and HRP2 with pLDH based rapid diagnostic tests for the diagnosis of malaria in middle Ghana, Africa
Source: PLoS One. 2018 Sep 7;13(9):e0203524. doi: 10.1371/journal.pone.0203524 (PMC6128572; doi:10.1371/journal.pone.0203524)
Supplement: S1 Table — (DOCX) [file pone.0203524.s001.docx]

**Supplementary Table Legend**

**S1 Table. Diagnostic performance of the *CareStart* malaria (HRP2) RDT kit to microscopy exploring parasite density cut-off points.**

| Correctly Cutpoint | Sensitivity | Specificity | Classified | Positive Likelihood Ratio (LR+) | Negative Likelihood Ratio (LR-) |
| --- | --- | --- | --- | --- | --- |
| >=25 | 100.00% | 0.00% | 98.22% | 1 |  |
| >=50 | 96.74% | 60.00% | 96.09% | 2.4185 | 0.0543 |
| >=100 | 93.12% | 100.00% | 93.24% |  | 0.0688 |
| >=200 | 86.23% | 100.00% | 86.48% |  | 0.1377 |
| >=1000 | 72.46% | 100.00% | 72.95% |  | 0.2754 |
| >=5000 | 58.70% | 100.00% | 59.43% |  | 0.413 |
| >=10000 | 50.36% | 100.00% | 51.25% |  | 0.4964 |
| >=50000 | 32.25% | 100.00% | 33.45% |  | 0.6775 |
| >50000 | 0.00% | 100.00% | 1.78% |  | 1 |
